# Researchers’ perspectives on pediatric obesity research participant recruitment

**DOI:** 10.1186/s40169-016-0099-0

**Published:** 2016-06-23

**Authors:** Yasha Parikh, Maryann Mason, Karen Williams

**Affiliations:** Central Michigan University College of Medicine, Mt Pleasant, MI USA; Northwestern University Feinberg School of Medicine, Chicago, IL USA; Center on Obesity Management and Prevention, Chicago, IL USA; Williams Heart Foundation, Northfield, IL USA

**Keywords:** Study recruitment, Pediatric obesity, Strategies, Qualitative

## Abstract

**Background:**

Childhood obesity prevalence has tripled over the last three decades. Pediatric obesity has important implications for both adult health as well as the United States economy. In order to combat pediatric obesity, exploratory studies are necessary to create effective interventions. Recruitment is an essential part of any study, and it has been challenging for all studies, especially pediatric obesity studies. The objective of this study was to understand barriers to pediatric obesity study recruitment and review facilitators to overcome recruitment difficulties.

**Methods:**

Twenty four childhood obesity researchers were contacted. Complete data for 11 researchers were obtained. Interviews were transcribed and analyzed using content analysis. Grounded Theory methodological approach was used, as this was an exploratory study. Investigators YP and MM coded the interviews using 28 codes.

**Results:**

Barriers to recruitment included: family and study logistics, family economics, lack of provider interest, invasive protocols, stigma, time restraints of clinicians, lack of patient motivation/interest, groupthink of students in a classroom, and participants who do not accept his or her own weight status. Facilitators to enhance recruitment practices included accommodating participants outside of regular clinic hours, incentivizing participants, cultivating relationships with communities, schools and clinics prior to study recruitment, emphasizing benefits of a study for the patient, and shifting language to focus on health rather than obesity.

**Conclusions:**

Pediatric obesity researchers face many standard and some unique challenges to recruitment, reflecting challenges common to clinical research as well as some specific to pediatrics and some specific to obesity research. Both pediatric studies as well as obesity studies are an added challenge to the already-difficult task of general study recruitment. Our findings can be used to make researchers more aware of potential difficulties, approaches and on-going needs for enhancing recruitment and enrollment practices, and in turn if applied, may result in increased study efficiency.

## Background

Over the last three decades, childhood obesity prevalence has more than tripled in the United States [1, 2]. Pediatric obesity is linked to disorders in every organ system, including cardiovascular disease, gastrointestinal conditions and endocrine disruption [3]. Children with obesity are also at increased risk of premature death, with a relative risk of 1.5 for all-cause mortality and 2.0 for mortality related to coronary heart disease [4]. The economic impact of childhood obesity is significant as well. Outpatient costs for children with obesity in 2010 surpassed $14 billion, and inpatient costs neared $240 million [5].

Although the research base related to pediatric obesity has been rapidly increasing, no one intervention has become a mainstay of therapy, which is troublesome given the scope of need [6–8]. Ideally, there would be a wide-range of evidence-based interventions available to treat and prevent childhood obesity given the different age, race/ethnicity groups, and community contexts affected by obesity. One fundamental barrier to the development of an intervention evidence-base is difficulty in enrolling children and their families in childhood-obesity-related research [9, 10].

Cooney et al. found that the challenge of convincing parents and children to enroll in studies is a major inhibitor to the development of an evidence base of effective interventions for reducing the prevalence of childhood obesity [11]. Gillespie et al. studied what prevented parents from enrolling their children in obesity studies, and potential answers included a lack of control, uncertainty, fear of confronting issues, and some just had no good reason [12]. Difficulty with recruitment is not specific to childhood obesity studies; increasing public participation in clinical trials was described as a central challenge facing the National Clinical Research Enterprise in 2003 [13].

Although recruitment is a known issue, the issue of pediatric recruitment and further, pediatric obesity study recruitment has yet to be well addressed in the literature. Current literature in this field defines what specific recruitment methods have proven to be most effective in terms of participation rates and cost-effectiveness, and even this literature is sparse [14–16]. Passive recruitment methods, such as newspaper advertisements or school newsletters, proved to be the most cost-effective and efficient recruitment method. Most studies had the most recruitment success when utilizing more than one recruitment method [16]. While these studies have a great deal of utility, they do not reflect actual experiences that pediatric obesity researchers have had with recruitment. Our goal with this study was to complement the existing quantitative literature with a qualitative study outlining actual researchers’ experiences with pediatric obesity research participant recruitment. By speaking to pediatric obesity researchers, we hoped to identify participant recruitment barriers for childhood obesity research in a range of research settings, and to inventory facilitators used by these researchers for enhancing recruitment outcomes. The ultimate goal is to inform future study recruitment efforts so that childhood obesity research recruitment outcomes can be improved and studies can be adequately powered and completed in a timely manner.

## Methods

This study is exploratory and used a cross-sectional qualitative design including semi-structured interviews to investigate experienced pediatric obesity researchers’ experiences with study recruitment. We used a Grounded Theory methodological approach for the study as it was exploratory in nature [17]. The Grounded Theory approach uses data to drive the construction of theory as opposed to other methodological approaches, which fit data to existing theoretical frameworks. The Grounded Theory methodological approach offers the advantage of discovery and theoretical development for areas that are under theorized and/or emergent. In this instance, we are trying to learn from experienced childhood obesity researchers to identify commonly experienced barriers and facilitators to achieving recruitment to develop data-informed conclusions about pitfalls to avoid and promising strategies to pursue regarding study participant recruitment activities.

### Participants and recruitment

Study participants were researchers working in clinical pediatric obesity research with firsthand experience in the recruitment of children with obesity. Researchers were required to have experience in recruitment to participate in the study, and this was explicitly asked in question 1 of the pre-screening questionnaire. Recruitment began by contacting all six center on obesity management and prevention (COMP) funded researchers, a group of researchers located in the Greater Chicago Area, where this study took place. These researchers provided names of others in the field, and the sample snowballed to 24. This recruitment strategy (contacting COMP researchers first) was utilized out of convenience due to the time and funding restraints of our research study. Geographically, our study represented two areas of the United States: the Greater Chicago area as well as the Northeast. Out of 24 researchers contacted for participation in our study, 13 (54 %) researchers agreed to speak with us. Of these, one was lost to follow-up and one did not meet the requirement of recruitment of children with obesity. Complete data were obtained for eleven researchers. Of the eleven researchers who did not participate, two refused participation and nine could not be reached.

### Interviews

Development of interview protocol began with a literature review to understand common study recruitment methods and factors affecting study recruitment strategies. Literature search terms included adolescent recruitment, child, epidemiology, health promotion, nutrition, obesity, overweight, parents/education, parents/psychology, participant recruitment, patient recruitment and pediatric recruitment. Existing literature was reviewed with a team of four researchers from COMP, who then utilized both the literature and personal experiences in recruitment to develop questions that highlighted the interviewees’ personal experiences. The interview protocol was piloted with three COMP researchers as a group for further refinement. These COMP researchers came from the same group of COMP researchers who assisted with recruitment for this study. COMP researchers who helped developed the interview protocol were not excluded from our study.

Each participant was asked a total of thirteen questions related to their specific experiences with recruitment in pediatric obesity studies, barriers, and strategies to overcome challenges within recruitment in pediatric obesity studies. The interview was conducted in a semi-structured, one-on-one format, allowing researchers to comment on personal experiences with recruitment methods. Ten separate interviews were conducted (two researchers were interviewed together because they participated in the recruitment for the same study). YP conducted nine interviews (six phone interviews and three face-to-face) while KW conducted one phone interview. Saturation was reached at interview seven, and no new themes emerged from an additional three interviews. Each interview lasted between thirty to sixty minutes. The interviews were digitally recorded and transcribed verbatim.

### Data analysis

Interview transcripts were analyzed using content analysis in which data were coded using a hierarchical organization of codes. A preliminary coding guide based on interview questions was created for coding of the first interview. Codes and coded segment groupings were revised using constant comparative methods in which previously coded data were compared to data undergoing coding to expand the understanding of all themes present within the data [18]. The first three transcripts were coded by two interviewers (YP and MM), and once agreement was reached between coders, one interviewer (YP) coded the remaining transcripts with assistance from MM. Analyses results were presented to the COMP research group for discussion and several refinements were made based on feedback. Refinements included adding clarity to theme names and theme descriptions. A total of 28 codes were created. All interview transcripts were re-coded with the final coding guide.

## Results and topics

### Characteristics of participants

Out of eleven participant-researchers, eight were female (73 %) and three were male (27 %). Eight participants had an MD degree (73 %), two held a PhD degree (18 %), and one held a Bachelor of Arts (9 %). Two researchers had exclusively school-based research experience (18 %) and six had exclusively clinical-based research experience (55 %). Two researchers had mixed experience with both clinical and community-based research (18 %), while another had mixed clinical and school-based research experience (9 %). Data about the studies described by researchers and individual recruitment methods utilized by the participants are listed in Table 1.

**Table 1:** Study settings and their respective recruitment methods reviewed during interviews

Study setting	Number of studies	Types of recruitment methods used	Study topic
Clinical	13	Clinical provider Posted flyers Online advertisement Email listserv RA recruitment in clinic	Nutrition Physical activity Imaging Genetics
Community	2	Email listserv Posted flyers Clinical provider	Physical activity
School	4	Brochure Opt-in consent worksheet Opt-out consent worksheet	Physical activity Nutrition

### Recruitment contexts

Three major recruitment contexts emerged from the interviews. Clinical recruitment included recruitment of eligible children with obesity by one or more physicians in a practice setting. School-based recruitment included recruitment done in the classroom via handouts or brochures given to students. Community-based recruitment involved recruitment done by direct contact between a researcher and a participant outside of a school or clinical setting, including emails, posted flyers in public settings such as daycares and boys and girls clubs, and online advertisements (Craigslist, Backpage). School-based recruitment is unique in that all children in a classroom, regardless of weight status, were recruited for research interventions, while clinical and community-based recruitment recruited only obese children.

### Barriers and strategies

**Fig. 1:**
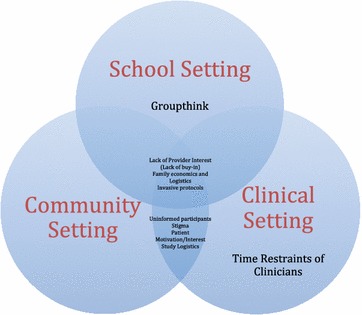
Barriers separated by context in which they commonly appear

Our results have been categorized based on setting (school, community, or clinic). Many barriers and strategies overlap between settings and this has been indicated where applicable (Fig. 1). Interestingly, there were no barriers specific to the community setting (individual recruitment). The barriers that did come up in the community setting also applied to both the clinical and school settings and have therefore been placed under the heading “*Barriers Represented in All Study Settings”*. A similar phenomenon occurred in facilitators; no facilitators were found in the community settings that did not also occur in either the school and clinical setting. Therefore, these facilitators were listed under the headings of “*Community and Clinical Setting” or “Facilitators in All Settings”*. Table 2 documents identified facilitators as they correlate with each barrier. Participant quotes are included in the results and represent a variety of participant-researchers.

**Table 2:** Barriers to recruitment and barrier-specific facilitators

Barriers	Facilitators
Study logistics	Provide transportation to and from clinic Provide parking for participants Provide compensation for self-travel Accommodate participants outside of work and school hours (weekends, evenings, holidays)
Family economics	Provide transportation to and from clinic Provide parking for participants Provide compensation for participants Provide an incentive for participation
Family logistics	Provide child-care for other children in family Accommodate participants outside of work and school hours (weekends, evenings, holidays)
Invasive protocols	Extensive risk vs. benefit discussion with participant and family Provide an incentive relative to the degree of invasive protocol
Lack of buy-in from recruitment site	Provide incentives for the recruitment site
Time restraint of clinicians	Provide incentives for the clinical site Provide personnel for a clinic specifically for research tasks
Stigma	Change the discussion from obesity-related language to health-promoting language
Uninformed participants or refusing to recognize obesity	Educate parents/caregivers and children about his or her own weight status Change the discussion from obesity-related language to health-promoting language
Groupthink	Buy-in from recruiters who will be presenting to the children (research personnel, school personnel, etc.)
Decreased patient motivation and/or interest	Extensive risk vs. benefit discussion with participant and family Change the discussion from obesity-related language to health-promoting language Provide incentives for participant and caregiver

### Barriers represented in all study settings

*Lack of recruitment site interest* (*lack of “buy-in”*) Researchers felt that there was a necessity for “buy-in” from the recruitment site. Buy-in is defined as on-site support of the research study through promoting information about the study to potential participants and their families and, in some cases, distributing participation information as instructed by the study manager.

- “When I went to the general pediatricians, there wasn’t a whole lot of ‘buy-in’. They were willing to help, but they really weren’t there to sell it.”
- “[Barriers included] whether or not you really got cooperation of teachers, whether or not the principal really bought-in, things of that nature… if we had a good cooperating physical education teacher identifying and encouraging kids, that made it pretty easy.”

*Family economics and logistics* Informants stated that family economics often caused hesitation for parents when considering participation in a study. Parking, gas, and time off of work negatively impacted family expenses and were often prohibitive for families. Having additional children who required care or were involved in other activities were also viewed as barriers to recruitment.

- “Our clinic recruits a lot of low income kids who often travel far from clinic and so we have found that the cost and burden of extra coming in has been a barrier”.
- “They may not have the funds to pay the gas to get there, or other things might be coming up that are more important to deal with as viewed by the family than driving the 2 h to the doctor, one-way”.

*Invasive protocols* Researchers felt that the more invasive protocols were less appealing to participants. Many parents worried about risks involved with invasive procedures. More invasive protocols were also more inconvenient for participants.

- “We’re trying to recruit obese and non-obese teenagers to measure blood pressure…They don’t want to have more lab work done other than the necessary lab work.”
- “Kids don’t want to participate because they don’t want the laboratory work done… [We’re] putting a 24 h cuff on them, and they don’t want that. They don’t like that. That’s the problem”.

### Barriers shared between the clinical and community settings

*Uninformed participants about weight status* Many parents and children were unaware of their weight status. This was mentioned as an issue in all recruitment settings and especially with passive recruitment– using flyers, ads, and email requiring interested participants to self-diagnose obesity.

- “One problem includes, doctor tells you you have to go see a nutritionist for your weight problem, you don’t believe you have a weight problem, so you don’t go”.
- “I did encounter several times where the parents seemed in denial about their child’s obesity and yes that wasn’t through clinic appointments, because the parents were accepting of that fact. That was through email.”

*Stigma* Stigma was discussed often by researchers. Both children and parents were affected by the negative connotation embodied in the words ‘obesity’ and ‘obesity study’, and this was reported as a deterrent for eligible patients participating in a study.

- “Some patients, they’re very sensitive about their weight, and it can be difficult. Since we also had controls in this study, I tried not to focus on the fact that the study was only for children with obesity, because we were also looking at healthy-weight children. I just said we were recruiting all types of children. It makes them more comfortable.”
- “It is hard for people to want to sign-up for something saying like, ‘oh yeah, my daughter is obese and I want to bring her in for the study’ rather than saying, ‘oh my daughter is healthy, lean, healthy weight’”.

*Patient motivation/interest* Many researchers felt that a large hindrance to recruitment came from a lack of patient motivation to comply with an unappealing intervention. Researchers commented on the unappealing nature of lifestyle changes such as nutrition therapy and physical activity changes as opposed to a more appealing ‘magic pill’. Researchers also commented on the fact that although patients may acknowledge his or her weight status, participants may not acknowledge that it is abnormal.

- “What we had to offer [the participants] wasn’t particularly appealing in the study. It wasn’t a medication, it was a lifestyle recommendation”.
- “This is a silent disease, so obese children have obese parents and nobody seems to be any worse for the wear at the time. Sot they’re not feeling ill, so they don’t feel the need to change anything”.

*Study logistics* Study logistics, such as available appointment times and inconvenient clinic locations were perceived as barriers to recruitment for most clinic and community researchers. Availability of clinic appointments was also seen as hindering recruitment as many clinical appointment slots were during school and work hours.

- “If they have to travel 2–3 h, depending on where they are, it could be more than 3 h, that’s a really big thing for people to do. It takes a lot of time and commitment and effort for people to do.”
- “We were not able to do the studies on the weekend because we did not have accessibility…so that is always difficult in school-aged kids. Typically, the summer-time was a good time to get all the kids in, but no, it was always during the week, and that was not always the most convenient for subjects.”

### Barriers specific to the clinical setting

*Time restraint of clinicians* Many researchers commented on the limited time clinicians have with patients as a barrier to recruitment. Remembering to recruit eligible patients, taking the time to discuss risks versus benefits, and collecting a thorough informed consent were not always feasible in the clinical setting.

- “Part of our biggest challenge is just remembering to even offer them an invitation. We are running around wildly during clinic time to see patients.”
- “People who design studies and expect a nurse in the practice to pull charts, or do extra measures, or talk to patients, or call patients—it’s just not going to happen. You have to respect private practice’s time.”
- “In the setting of a busy clinic, you have 1000 things on your mind, and the last thing you’re thinking of is recruiting for someone else’s study”.

### Barriers specific to the school setting

*Groupthink* An important consideration mentioned by researchers engaged in school-based research was the idea of groupthink—where one child made a decision and those children in close proximity to that child automatically made the same decision. This was perceived as a barrier to recruitment for school-researchers who spoke to entire classrooms of children at the same time.

- “One kid will be sitting in the corner saying, ‘I’m not doing this, this is stupid’ and the next thing you know, the six kids around him will be doing the same thing”.

### Facilitators for recruitment in all study settings

*Incentives* Researchers highlighted the importance for incentives to successful recruitment. Three different levels of incentives were utilized: incentives for the participant, incentives for the caretakers (e.g. family), and incentives for the clinics.

- **“**I’ve been in two studies…and in one study, we provided a practice-level incentive of $1000 each time they had to do a set of measures or help us with something… and the other, we didn’t have the money to provide much, and there was definitely a difference [in recruitment].”
- “Seeing a colleague that worked with a similar age range, where [the participants] had to do much more, but were given more money on the gift card, recruitment was just so much easier for them”.
- “Incentives really work, so a lot of studies will use a really modest incentive with an incentive for both the subject and the caregiver, acknowledging that it takes their time and gas to bring their kid in, so we give something to both parties”.

*Pre-existing relationships* Many researchers commented on the usefulness of pre-existing relationships with clinics, schools, and community leaders. Three researchers commented on the success of having pre-existing relationships with clinics, and many agreed that relationships in all settings require time to cultivate, but can improve recruitment down the line.

- “My experience is that a lot of [recruitment] is driven by the study coordinator who develops relationships with the school administrators, and with the teachers, and with the school, and if that study coordinator has the ability to do so, has the personality for it, things seemed to go relatively smoothly, and if not, you’ll be in trouble”.
- “I mean, it would have been faster for us if we had just established those relationships with the clinic immediately, so I would just suggest before you start a study, talk about that study with other clinics like an endocrinology clinic or an obesity clinic or the like, so you don’t have any lag time”.
- “I have a very good relationship with the doctor, the director of the clinic. Once she was interested, once she knew about the criteria for recruiting, she was telling her patients. So what facilitated the study was the doctor talking about the study with the patients.”

*Identifying benefits of a study* Researchers emphasized the importance of framing a discussion of study benefits to include personal benefits to help participants become more motivated about enrolling in a study. Researchers also were careful to build in personal benefits to study participation when they could. These strategies also were also perceived to help reduce or eliminate concerns about stigma.

- “You want to make sure your study design isn’t in any way adding to stigma; you want to make sure all of your materials are worded appropriately, non-judging, and are not identifying a problem but offering a solution”.
- “Attrition for obesity treatment is so high, it’s hard to get people coming in [to the clinic] that…is true for obesity studies as well, so the more you can incorporate it into regular care, not call more attention to it, [the better]”.
- “There was blood studies associated with the medical nutrition therapy study, and the thing of value there was…’here are your health parameters for free’…showing the subject or subject’s family what the benefit of participating [is]”.

### Facilitators in the community and clinic settings

*Accommodations* Being more flexible regarding participation requirements was seen as boosting recruitment results. Accommodations mentioned include providing child-care, more clinic availability, including weekends and holidays, utilizing less invasive, more “in-dwelling” procedures (e.g. blood draws done in the home, etc.), and using telephone or computer data collection (versus face-to-face). Accommodations for clinics were also seen as important, including providing research personnel in the clinic to take care of all research-related work.

- “Accommodations that we made were that we had appointments after school hours and weekends. That helped a ton. Particularly weekends …there’s at least 5-6 patients that you could see if it was just not working in the work week”.
- “We had straggler busses, if you will. In every group, there were always people who were sick or whatever. We just tried to pick them up the next day, next week, what have you”.
- “What we did was that we had the cooperation of other people in the clinic, so they stayed with the other members of the family if they needed to bring their kids. We offered for them to be with them to kind of not directly care for these kids, but be with them while the other part of the family was with the other kids”.

### Facilitators in the school setting

*Passive recruitment (opt-out vs. opt-in)* Seen as especially important in the school setting, researchers embraced the opt-out consent strategy as effective for study recruitment. This involved obtaining a parent signature to exclude a child from a study instead of requiring a signature for inclusion in a study.

- “With the opt-out procedure, which is much easier on the school because there is much less paperwork to collect, we can get a 90–95 % success rate in recruitment”.
- **“**We have had bad experiences, or less than successful experiences, getting children’s parents to provide active consent to participate in a study or an evaluation. Then we worked with the research review board and the [school system] to get passive consent…that has greatly increased our participation. Something around 80–90 % [students] participate in that scenario.”

## Discussion

The Agency for Healthcare Research and Quality (AHRQ) notes that achieving adequate participant recruitment is a significant challenge in many research studies. A recent survey reported by AHRQ found that 34 % of studies participating in the survey recruited less than 75 % of their planned sample, leading to reductions in the statistical power of studies [19].

Barriers to research participation from the perspective of patients are widely acknowledged. A National Cancer Institute (NCI) synthesis report explored barriers to clinical trial participation from the patient perspective and found over 20 different barriers to enrollment including: patient fears of being experimented on, costs, quality of life concerns, logistical concerns, effort involved in the informed consent process, preference for alternative treatments, beliefs about the ineffectiveness of treatments, and concerns about continuity in care [20].

Another study examining barriers to participation specific to childhood obesity programs and research in the Netherlands from the youth health worker perspective found that parental denial or lack of awareness of obesity as a problem facing children, normalization of obesity in society, transportation, and parental concerns about the intervention were perceived as leading barriers to participation [21]. A notable difference between these two studies is that the study reporting perspectives of health care workers did not identify the acceptability of the research or intervention from the patient and family perspective as a main concern while the NCI study from the patient perspective did. This suggests that researchers have less awareness of significant patient concerns regarding research participation.

Our study, which reports researcher’s perspective on barriers and facilitators to childhood obesity research participation, has findings similar to the study reporting findings from the youth health care worker perspective in that it finds that researchers emphasized logistical barriers while perhaps under-reporting on the influence of the acceptability of the research or intervention to patients and families. This suggests that there may be a need to explore how well researchers understand the types of interventions that are acceptable to patients and families when they are planning research projects.

Our study also found that participating researchers had experimented with addressing barriers to study participation, using different approaches both during and between studies in which they were involved. Most of these efforts were identified by participating researchers as facilitators to research participation and included efforts to reduce participant burdens including realistically compensating transportation, conducting research in-dwelling when possible, and improving appointment convenience. This indicates that some degree of flexibility in the approach to study recruitment is likely necessary to maximize participation during the planning and implementation stages of research.

No researchers in our study discussed efforts to address patient and parents concern about obesity as a health problem or increase recognition of obesity in children as ways to facilitate research participation, though these were identified as barriers to study participation by most researchers in our study. This indicates that these may be areas for further consideration and research as the field of childhood obesity research moves forward.

Beyond the development of study participation processes that minimize participant burden, participants in our study also identified the development of trusting relationships with clinical care providers who may refer and/or enroll study participants and community and school representatives who can facilitate the distribution of recruitment materials and serve as a trusted source of information among potential participants in non-clinical settings as facilitating research recruitment and participation. While researchers participating in our study did not elaborate on this, there are well-established resources for partnership development utilizing relationships in the community, schools, and clinics is a central focus of the Patient-Centered Outcomes Research Institute (PCORI), which has begun to fund such studies as well as work in community engaged research (CEnR) which could serve as resources for this [22, 23].

In fact, many of the facilitators to recruitment identified by researchers in our study can be considered practices of ‘patient-centeredness’, and community engaged scholarship including developing and maintaining relationships with community/school/clinic leaders, appropriately identifying study benefits for the patient, and accommodations tailored to the participant. The approach is an essential part of the Affordable Care Act, put in place to fund patient-centered research (as opposed to comparative clinical effectiveness research). This approach is different from the classic clinical effectiveness approach in that this approach takes into consideration the preferences of participants rather than the more standard preference of the researcher [24]. Patient-centered and community-engaged scholarship approaches have emerged as promising practices in health research [25].

Strengths of our study include assessing recruitment strategies specifically designed for obesity-related research. This is useful because there are many barriers to recruitment distinctly related to obesity, such as denial, stigma, and a lack of patient motivation that may not be present in other study areas. Finally, our study evaluated actual actions taken by researchers versus future plans for recruitment.

Our study also had several limitations. One limitation is the use of convenience based snowball sampling. This resulted in limited geographic representation (Northeast U.S. and Chicago are well represented, other areas of the U.S. were not). Another limitation may be the use of self-reported recall information versus information from IRB applications, which may include more thorough information. A third limitation in our study is that interviews were conducted in two ways (by phone and in person). Participants may have responded differently face-to-face instead of over the phone, and this may have introduced another variable in our study. Thirdly, it would have been beneficial to track recruitment goals versus results to evaluate outcomes using standardized measures. Fourthly, many of our barriers had overlaps (such as a lack of buy-in from a provider leading to a lack of motivation on the part of the participant). Lastly, the interviews did not touch on well-defined barriers in the literature, and instead focused on individual experiences with recruitment. Perhaps future studies can address researchers discussing his or her own experience with well-described barriers and facilitators specifically addressing these barriers to recruitment.

In conclusion, pediatric obesity researchers face many standard and some unique challenges to recruitment, reflecting challenges common to clinical research as well as some specific to pediatrics (need for family involvement, competing child activities) and some specific to obesity research (e.g. recognition of obesity in child, recognition of obesity as a health problem). Both pediatric studies as well as obesity studies are an added challenge to the already-difficult task of general study recruitment. These findings can be used to make researchers more of aware of potential difficulties, approaches, and on-going needs for enhancing recruitment and enrollment practices, and in turn, if applied, may result in increased study efficiency.

## Abbreviations

AHRQ: agency for healthcare research and quality
CEnR: community engaged research
COMP: center on obesity management and prevention
IRB: institutional review board
NCI: national cancer institute
PCORI: patient-centered outcomes research institute
